# Associations of short sleep duration with prehypertension and hypertension among Lithuanian children and adolescents: a cross-sectional study

**DOI:** 10.1186/1471-2458-14-255

**Published:** 2014-03-15

**Authors:** Renata Kuciene, Virginija Dulskiene

**Affiliations:** 1 Laboratory of Population Studies, Institute of Cardiology, Medical Academy, Lithuanian University of Health Sciences, Sukileliu 17, LT-50009 Kaunas, Lithuania

**Keywords:** Sleep duration, Prehypertension, Hypertension, Children, Adolescents

## Abstract

**Background:**

Recent epidemiological studies have found that the prevalence of high blood pressure (BP) has significantly increased among children and adolescents. The aim of this study was to examine the associations between short sleep duration and prehypertension and hypertension in Lithuanian children and adolescents aged 12 to 15 years.

**Methods:**

A cross-sectional study was conducted from November 2010 to April 2012. The participants with high BP (≥90th percentile) were screened on two separate occasions. Self-reported sleep duration was evaluated using questionnaires. Data on 6,940 subjects aged 12–15 years were analyzed. Adjusted odds ratios (aORs) with 95% confidence intervals for the associations were estimated using multivariate logistic regression models. Short sleep duration was defined as <8 hours per day (h/day).

**Results:**

The prevalence of prehypertension and hypertension in the current sample was 12.6% and 22.5%, respectively. The percentages of the subjects with sleep durations of <7 (h/day), 7– < 8 h/day, and ≥8 h/day were 8.7%, 21.0%, and 70.3%, respectively. After adjusting for age, sex, body mass index, physical activity, and smoking, significant associations were found between short sleep duration and high BP, including prehypertension (7– < 8 h/day: aOR = 1.77; 95% CI, 1.48–2.12; <7 h/day: aOR = 2.18; 95% CI, 1.70–2.79) and hypertension (7– < 8 h/day: aOR = 1.99; 95% CI, 1.72–2.31; <7 h/day: aOR = 2.28; 95% CI, 1.85–2.80) (all P values <0.001), compared to participants who were sleeping longer (≥8 h/day).

**Conclusions:**

Prehypertension and hypertension were associated with short sleep duration among Lithuanian children and adolescents aged 12 to 15 years.

## Background

Epidemiological studies have reported an increased prevalence of high blood pressure (BP) among children and adolescents in various countries of the world during the recent years [1-9]. It has been demonstrated that BP tracks significantly from childhood and adolescence into adulthood [10]. It is well known that high BP, or hypertension, in adults increases the risk of cardiovascular diseases [11-13]. According to the World Health Organization, high BP is the leading risk factor affecting mortality (7.5 million (13%) of all deaths globally per year) and disability-adjusted life years worldwide [14].

Sleep plays an essential role in growth, maturation, and health during childhood and adolescence [15]. Reduced sleep duration and poor quality of sleep are common problems among children and adolescents [16-19]. The National Sleep Foundation Survey has reported that the average sleep duration on school nights among US adolescents (age 11–17) was 7.6 hours; furthermore, 45% of adolescents got an insufficient amount of sleep (less than 8 hours) [19]. The HELENA (Healthy Lifestyle in Europe by Nutrition in Adolescence) study has indicated that about 33% of adolescents aged 12.5-17.5 years from ten European cities reported sleeping <8 hours per day (h/day) [20]. Insufficient sleep can be caused by interaction between extrinsic factors (e.g. social, environmental, behavioral, and other) and intrinsic factors (e.g. puberty) [17].

Growing scientific evidence supporting the associations between short sleep duration and hypertension, or high BP among adults has been described and summarized in recent reviews [21,22]. Few studies have investigated the association between sleep duration and high BP among children and adolescents [3,5,23-27]; however, the results of these studies have been inconsistent. Therefore, it is becoming increasingly important to identify potential risk factors associated with an increased risk of cardiovascular diseases and other chronic non-communicable diseases with a particular focus on modifiable risk factors among children and adolescents, and then to plan, develop, and implement clinical and public health strategies for the prevention of these diseases.

The association between short sleep duration and high BP among children and adolescents has not been studied in Lithuania before. Moreover, the prevalence of high BP (hypertension) in Lithuanian children [28] and adult populations [29,30] is very high. Cardiovascular diseases in our country are the most common cause in the structure of causes of death, and mortality from these diseases remains one of the highest in Europe [31].

Thus, the aim of this study was to evaluate the associations between short sleep duration and prehypertension and hypertension among children and adolescents living in Kaunas city and in Kaunas district (Lithuania).

## Methods

### Study population

A cross-sectional study was performed, continuing from November 2010 to April 2012. This study included subjects aged 12 to 15 years who at the time of the examination attended gymnasiums or secondary schools in Kaunas city and Kaunas district - the second largest city and district by population size in Lithuania. All the invited schools (n = 81) accepted the invitation to participate in the research project.

Of 7,638 subjects who participated and were examined in the study, 152 subjects were excluded from the statistical analyses because they had any of the following diseases: endocrine diseases, diabetes mellitus, kidney diseases, cardiovascular diseases, or congenital heart defects (information was collected from the subjects’ medical records (Form No.027-1/a)). In addition, 29 subjects were excluded due to missing data on weight and height. In addition, data on 517 participants were excluded from the analysis because of incomplete information about sleep duration, physical activity, and smoking. Thus, data on 6,940 participants were approved for statistical analysis.

Both BP and anthropometric measurements were performed at the participants’ schools by the same team of trained study personnel (physicians and research assistants). After measurements, all subjects were asked to fill out the questionnaires. The standard questionnaires included information on selected risk factors, such as sleep duration, physical activity, smoking status, etc.

A written informed consent was obtained from each participant’s parent or guardian. The study was approved by the Kaunas Regional Ethics Committee for Biomedical Research at the Lithuanian University of Health Sciences (protocol No. BE-2-69).

### Measurements

#### Blood pressure measurement

Blood pressure was measured by the physician who did not wear a white coat in the morning hours (8:30 am to 11:30 am). The participants were advised to avoid energy drinks, coffee, tea, and physical exercises on the morning of the study day until the measurement. Before the BP measurement, the subjects were asked to sit still for ten minutes. BP was measured three times with a 5-minute rest interval between the measurements, with the participant in a sitting position, and using an automatic BP monitor (OMRON M6; OMRON HEALTHCARE CO., LTD, Kyoto, Japan). The mean of three BP measurements was calculated and used for the analysis. All subjects with high BP (BP ≥90th percentile) at the first screening underwent the second screening of BP measurements within the period of 2–3 weeks.

Classifications and definitions of BP levels were defined according to the guidelines of “The Fourth Report on the Diagnosis, Evaluation, and Treatment of High Blood Pressure in Children and Adolescents” (National High Blood Pressure Education Program (NHBPEP) Working Group on High Blood Pressure in Children and Adolescents) [32]. According to BP charts for age, sex, and height, normal BP was defined as systolic blood pressure (SBP) and diastolic blood pressure (DBP) below the 90th percentile; prehypertension was defined as average SBP or DBP levels equal to or above the 90th percentile, but below the 95th percentile; and hypertension was defined as mean SBP or DBP equal to or above the 95th percentile.

#### Anthropometric measurement

Height and weight of the subjects were measured with the accuracy of 0.1 cm and 0.1 kg, respectively, by using a portable stadiometer and a balance beam scale. The participants were barefoot and wearing light clothes. Body mass index (BMI) was calculated as weight in kilograms divided by the square of height in meters (kg/m^2^). According to the cut-off points of BMI proposed by Cole et al. [33], the participants were grouped into three categories of BMI: normal weight, overweight, and obese.

### Data determined using the questionnaire

The questionnaires recorded information on selected risk factors, such as sleep duration, physical activity, smoking status, etc.

Sleep was determined using the question “How many hours do you usually sleep per day on the average (including sleep at night and during the day)?” Sleep duration was categorized into three groups: <7 h/day; ≥7 and <8 h/day; and ≥8 h/day (reference category). Short sleep duration was defined as sleeping less than 8 h/day.

The participants were asked how many hours they were physically active (including walking or cycling to and from school and during leisure time; and sports activities during physical education lessons and during leisure time). The participants were categorized into three groups according to their physical activity: <0.5 h/day, 0.5– < 1 h/day, and ≥1 h/day.

Current smoking status was categorized into “no” or “yes”. The participants who smoked at least one cigarette per day were classified as current smokers.

### Statistical analysis

Comparisons between the groups were performed by applying the chi-squared (*χ*^2^) test (for categorical variables), Mann–Whitney *U* test and Kruskal-Wallis test (for non-normally distributed continuous variables), and *t*-test and ANOVA (for normally distributed continuous variables). Descriptive statistics (mean and standard deviation (SD)) were calculated for the quantitative variables (age, weight, height, BMI, SBP, and DBP). Univariate and multivariate logistic regression analyses were conducted separately for boys and girls and for both sexes combined to evaluate the associations of short sleep durations with prehypertension and hypertension. Crude odds ratios (ORs) and adjusted odds ratios (aORs) along with 95% confidence intervals (CIs) were calculated. In multivariate analysis for boys and girls separately, ORs were adjusted for age, BMI, physical activity, and smoking. In the multivariate analysis for both sexes combined, two models were performed: in the first model, ORs were adjusted for age and sex; in the second – the final model, ORs were adjusted for age, sex, BMI, physical activity, and smoking.

Statistical analyses were performed using the statistical software package SPSS version 20 for Windows. P values <0.05 were considered statistically significant.

## Results

Table 1 shows the characteristics of the study population. Among the 6,940 study participants aged 12–15 years, 46.0% (n = 3,189) were boys, and 54.0% (n = 3,751) were girls. The mean age of all participants was 13.44 ± 1.11 years (boys: 13.43 ± 1.12 years, girls: 13.44 ± 1.10 years; no significant difference in age was found between these groups (P = 0.852)). Boys were significantly taller, heavier, and had a higher BMI. They also had a significantly higher mean SBP and a significantly lower mean DBP than girls did.

**Table 1:** Demographic, anthropometric, and BP characteristics of the study participants by sex; values are mean ± SD

**Variables**	**Total (n = 6940)**	**Boys (n = 3189)**	**Girls (n = 3751)**	P^*^
Age (years)	13.44 ± 1.11	13.43 ± 1.12	13.44 ± 1.10	0.852
Height (cm)	164.62 ± 9.89	166.70 ± 11.44	162.84 ± 7.94	<0.001
Weight (kg)	53.40 ± 11.93	55.14 ± 13.05	51.87 ± 10.60	<0.001
BMI (kg/m^2^)	19.56 ± 3.19	19.65 ± 3.14	19.45 ± 3.21	<0.001
SBP (mmHg)	118.31 ± 14.43	121.89 ± 15.99	115.27 ± 12.15	<0.001
DBP (mmHg)	65.59 ± 7.76	65.15 ± 7.84	65.96 ± 7.67	<0.001

*BP* blood pressure, *BMI* body mass index, *SBP* systolic blood pressure, *DBP* diastolic blood pressure.

^*^Boys versus girls (Mann–Whitney *U* test).

Overall, the prevalence of prehypertension and hypertension was 12.6% (n = 875) and 22.5% (n = 1561), respectively. The prevalence of prehypertension and hypertension was significantly higher in boys (14.9% and 29.6%, respectively) than in girls (10.7% and 16.4%, respectively). A higher prevalence of prehypertension and hypertension was identified in the participants aged 14–15 years than aged 12–13 years (43.3% versus 27.4%). Overall, 21.0% of the participants (18.1% of boys and 23.4% of girls) reported sleep duration of 7– < 8 h/day, and 8.7% of the subjects (8.2% of boys and 9.1% of girls) reported sleep duration of <7 h/day. Among subjects with short sleep durations of 7– < 8 h/day and <7 h/day, 16.1% and 18.5% had prehypertension, respectively, and 29.2% and 31.5% had hypertension, respectively. In total, the prevalence of overweight and obesity was 12.0% (14.1% for boys and 10.3% for girls) and 2.4% (2.6% for boys and 2.2% for girls), respectively. Only 8.5% of all participants (12.5% of boys and 5.2% of girls) reported physical activity of ≥0.5–1 h/day, and 2.4% of the participants (3.0% of boys and 1.9% of girls) reported physical activity of <0.5 h/day. Overall, 7.8% of the participants (8.7% of boys and 7.1% of girls) were current smokers.

Table 2 presents the general characteristics of the study participants according to the BP level. All risk factors (shorter sleep duration, BMI status (overweight and obesity), lower physical activity, and current smoking) were more prevalent among prehypertensive and hypertensive participants than among normotensives. Prehypertensive and hypertensive subjects had significantly higher mean values for age, weight, height, BMI, SBP, and DBP, compared to those with normal BP.

**Table 2:** Characteristics of the study participants according to blood pressure levels

**Variables**	**Normotensive (n = 4504)**		**Prehypertensive (n = 875)**		**Hypertensive (n = 1561)**		P*
**Categorical variables**^¶^	**n**	**%**	**n**	**%**	**n**	**%**	
Sex:							
Boys	1,770	39.3	475	54.3	944	60.5	<0.001
Girls	2,734	60.7	400	45.7	617	39.5	
Age (years):							
12–13	2,608	57.9	292	33.4	694	44.5	<0.001
14–15	1,896	42.1	583	66.6	867	55.5	
Sleep duration (h/day):							
≥8	3,406	75.6	529	60.5	947	60.6	<0.001
7– < 8	796	17.7	234	26.7	424	27.2	
<7	302	6.7	112	12.8	190	12.2	
BMI categories:							
Normal weight	4,108	91.2	691	79.0	1,140	73.0	<0.001
Overweight	349	7.8	149	17.0	337	21.6	
Obesity	47	1.0	35	4.0	84	5.4	
Physical activity (h/day):							
≥1	4,066	90.3	762	87.1	1,351	86.5	<0.001
0.5– < 1	339	7.5	85	9.7	169	10.8	
<0.5	99	2.2	28	3.2	41	2.7	
Smoking:							
No	4,171	92.6	782	89.4	1,444	92.5	0.003
Yes	333	7.4	93	10.6	117	7.5	
**Continuous variables**^**#**^	**mean ± SD**		**mean ± SD**		**mean ± SD**		P**
Age (years)	13.29 ± 1.09		13.89 ± 1.03^a^		13.62 ± 1.09^a,b^		<0.001
Weight (kg)	49.84 ± 10.15		59.54 ± 11.31^a^		60.22 ± 12.66^a^		<0.001
Height (cm)	162.37 ± 9.32		169.75 ± 8.87^a^		168.20 ± 9.90^a,***b***^		<0.001
BMI (kg/m^2^)	18.77 ± 2.76		20.59 ± 3.22^a^		21.17 ± 3.50^a,b^		<0.001
SBP (mmHg)	109.72 ± 6.93		124.85 ± 3.90^a^		139.41 ± 9.85^a,b^		<0.001
DBP (mmHg)	63.10 ± 6.38		67.22 ± 6.91^***a***^		71.85 ± 8.08^***a,b***^		*<0.001*

^¶^Values are numbers and percentages.

^#^Values are means ± SD (standard deviations).

*BMI* body mass index, *SBP* systolic blood pressure, *DBP* diastolic blood pressure.

*Significant differences between the groups were determined by the chi-squared (*χ*^2^) test.

**Significant differences between the three groups were determined by Kruskal-Wallis test and ANOVA (data in italics).

^a^Significantly different (P < 0.05) from normotensive participants (Mann–Whitney *U* test; *t*-test).

^b^Significantly different (P < 0.05) from prehypertensive participants (Mann–Whitney *U* test; *t*-test).

^***a,b***^Superscript letters in bold and italics indicate that significant differences (P < 0.05) between groups were determined by the *t*-test.

Table 3 shows the characteristics of the subjects by sleep duration categories. The participants with short sleep durations (7– < 8 h/day and <7 h/day) had significantly higher mean values for age, weight, height, BMI, SBP, and DBP, compared with the participants who slept ≥8 h/day. Among girls, the means of SBP, DBP were significantly higher in the group sleeping <7 h/day than in the group sleeping 7– < 8 h/day, but for boys, these differences were not statistically significant.

**Table 3:** Characteristics of the study participants according to sleep duration categories; values are mean ± SD

**Variables**	**<7 h/day**	**7– < 8 h/day**	**≥8 h/day**	P*
***Boys:***	**(n = 261)**	**(n = 576)**	**(n = 2352)**	
Age (years)	13.74 ± 1.12^a^	13.69 ± 1.08^a^	13.35 ± 1.11	<0.001
Weight (kg)	58.34 ± 12.99^a^	57.78 ± 12.53^a^	54.24 ± 13.07	<0.001
Height (cm)	169.30 ± 11.92^a^	168.88 ± 10.79^a^	165.88 ± 11.43	<0.001
BMI (kg/m^2^)	20.16 ± 3.01^a^	20.08 ± 3.06^a^	19.49 ± 3.16	<0.001
SBP (mmHg)	124.54 ± 15.32^a^	126.47 ± 15.93^a^	120.47 ± 15.84	<0.001
DBP (mmHg)	65.87 ± 8.43^a^	66.18 ± 8.23^a^	64.83 ± 7.65	<0.001
***Girls:***	**(n = 343)**	**(n = 878)**	**(n = 2530)**	
Age (years)	13.75 ± 1.11^a^	13.74 ± 1.05^a^	13.29 ± 1.08	<0.001
Weight (kg)	55.61 ± 11.13^a,b^	53.96 ± 10.49^a^	50.60 ± 10.39	<0.001
Height (cm)	165.07 ± 6.56^a^	164.17 ± 7.92^a^	162.08 ± 8.00	<0.001
BMI (kg/m^2^)	20.33 ± 3.53^a^	19.93 ± 3.09^a^	19.16 ± 3.17	<0.001
SBP (mmHg)	119.84 ± 13.07^a,b^	117.38 ± 12.54^a^	113.91 ± 11.62	<0.001
DBP (mmHg)	68.62 ± 8.26^a,b^	66.97 ± 7.85^a^	65.24 ± 7.41	<0.001
***All participants:***	**(n = 604)**	**(n = 1454)**	**(n = 4882)**	
Age (years)	13.74 ± 1.12^a^	13.72 ± 1.06^a^	13.32 ± 1.10	<0.001
Weight (kg)	56.79 ± 12.03^a,b^	55.47 ± 11.49^a^	52.36 ± 11.89	<0.001
Height (cm)	166.90 ± 9.49^a^	166.03 ± 9.45^a^	163.91 ± 9.98	<0.001
BMI (kg/m^2^)	20.26 ± 3.32^a^	19.99 ± 3.08^a^	19.32 ± 3.17	<0.001
SBP (mmHg)	121.87 ± 14.27^a^	120.98 ± 14.67^a^	117.07 ± 14.20	<0.001
DBP (mmHg)	67.43 ± 8.44^a,b^	66.66 ± 8.01^a^	65.04 ± 7.53	<0.001

*SD* standard deviation, *BMI* body mass index, *SBP* systolic blood pressure, *DBP* diastolic blood pressure.

^a^Significantly different (P < 0.05) from group of ≥8 h/day (Mann–Whitney *U* test).

^b^Significantly different (P < 0.05) from group of 7– < 8 h/day (Mann–Whitney *U* test).

*Differences between the three groups were determined by Kruskal-Wallis test.

The results of univariate and multivariate analyses are presented in Table 4 (separately for both sexes) and Table 5 (for both sexes combined).

**Table 4:** Associations between sleep durations and prehypertension and hypertension by sex (univariate and multivariate analyses)

**Variables**	**Normal BP**		**Prehypertension**				**Hypertension**			
	**n**	**%**	**n**	**%**	**OR (95% CI)**	**aOR**^**1 **^**(95% CI)**	**n**	**%**	**OR (95% CI)**	**aOR**^**1 **^**(95% CI)**
***Boys:***										
Sleep duration (h/day):										
≥8	1424	80.5	316	66.5	1.00	1.00	612	64.8	1.00	1.00
7– < 8	229	12.9	112	23.6	2.20 (1.71–2.85)	1.86 (1.41–2.44)	235	24.9	2.39 (1.95–2.93)	2.13 (1.72–2.65)
<7	117	6.6	47	9.9	1.81* (1.26–2.59)	1.49* (1.01–2.19)	97	10.3	1.93 (1.45–2.57)	1.70* (1.26–2.31)
***Girls:***										
Sleep duration (h/day):										
≥8	1982	72.5	213	53.3	1.00	1.00	335	54.3	1.00	1.00
7– < 8	567	20.7	122	30.4	2.00 (1.57–2.55)	1.81 (1.41–2.32)	189	30.6	1.97 (1.61–2.41)	2.00 (1.62–2.46)
<7	185	6.8	65	16.3	3.27 (2.38–4.48)	3.01 (2.17–4.18)	93	15.1	2.97 (2.26–3.92)	3.07 (2.31–4.09)

OR – crude odds ratio; aOR^1^ – adjusted odds ratios for age, body mass index, physical activity, and smoking; CI – confidence interval.

All results were significant at P < 0.001, except when noted (* – P < 0.05).

**Table 5:** Associations between sleep durations and prehypertension and hypertension for both sexes combined (univariate and multivariate analyses)

**Variables**	**Prehypertension**			**Hypertension**		
	**OR**	**aOR**^**1**^	**aOR**^**2**^	**OR**	**aOR**^**1**^	**aOR**^**2**^
	**(95% CI)**	**(95% CI)**	**(95% CI)**	**(95% CI)**	**(95% CI)**	**(95% CI)**
Sleep duration (h/day):						
≥8	1.00	1.00	1.00	1.00	1.00	1.00
7– < 8	1.89	1.80	1.77	1.92	2.01	1.99
	(1.59–2.25)	(1.50–2.15)	(1.48–2.12)	(1.67–2.20)	(1.74–2.32)	(1.72–2.31)
<7	2.39	2.21	2.18	2.26	2.25	2.28
	(1.89–3.02)	(1.73–2.81)	(1.70–2.79)	(1.86–2.75)	(1.84–2.76)	(1.85–2.80)

OR – crude odds ratio; aOR^1^ – adjusted odds ratios for age and sex; aOR^2^ –adjusted odds ratios for age, sex, body mass index, physical activity, and smoking; CI – confidence interval.

All results were significant at P < 0.001.

Univariate analysis revealed that short sleep durations (7– < 8 h/day and <7 h/day) were significantly associated with higher odds of prehypertension and hypertension for both sexes separately (Table 4) as well as for the combined group consisting of both boys and girls (Table 5), compared to the participants who reported sleeping ≥8 h/day.

As shown in Table 4, after adjusting for age, BMI, physical activity, and smoking, boys and girls with shorter sleep duration were at higher odds of elevated BP as compared to those sleeping ≥8 h/day. Sleep durations 7– < 8 h/day and <7 h/day were associated with higher odds of both prehypertension (among boys: aOR = 1.86 and aOR = 1.49, respectively, and among girls: aOR = 1.81 and aOR = 3.01, respectively) and hypertension (among boys: aOR = 2.13 and aOR = 1.70, respectively, and among girls: aOR = 2.00 and aOR = 3.07, respectively).

For both sexes combined, adjustments for potential confounding factors in the first and the second models did not affect the significance of the associations between short sleep duration and high BP, although the aORs changed a little (Table 5). According to the final models, sleep duration of <7 h/day was associated with higher odds of prehypertension (aOR = 2.18) and hypertension (aOR = 2.28) (both P < 0.001), if compared with sleep duration of ≥8 h/day. Sleep duration of 7– < 8 h/day was also significantly associated with prehypertension (aOR = 1.77) and hypertension (aOR = 1.99) (both P < 0.001).

## Discussion

To our knowledge, this is the first report that investigated the association between short sleep duration and high BP among 12–15 year-old children and adolescents in Lithuania. Univariate and multivariate logistic regression analyses (for both sexes separately and combined) of our data showed significant associations between short sleep durations (7– < 8 h/day and <7 h/day) and prehypertension and hypertension among children and adolescents.

The results of the current study indicated a high prevalence of elevated BP (prehypertension: 12.6% and hypertension: 22.5%) among children and adolescents, and these findings are partially consistent with the findings of other previously published studies [3,5,34,35].

With regard to sleep duration, there is no accurate consensus on the cut-off values of short, optimal, and long sleep durations among children and adolescents. Nevertheless, according to the guidelines of the National Sleep Foundation, the amount of sleep per night for adolescents is insufficient if <8 hours, borderline if 8 to <9 hours, and optimal if ≥9 hours [19]. It has also been suggested that adolescents aged 10–17 years need 8.5-9.25 hours of sleep per night [36]. Based on the above-mentioned definition of insufficient sleep [19], about one-third of the participants (29.7%) in the present study reported insufficient or short sleep duration (<8 h/day). Our finding regarding the high prevalence of insufficient sleep are consistent with the findings of other studies done on almost similar age groups of adolescents [19,20]. The comparison of the results of the associations from the present study with the results from other previous studies is complicated because there are differences in the sample size, the age of the included children and adolescents, the number of BP measurements, the measurement of sleep duration, cut-off values for defining short sleep duration, and adjustments for different confounders. Furthermore, previous studies have reported different results. For example, a cross-sectional study in China found that parent-reported short sleep duration (<9 hours) was significantly associated with hypertension (aOR = 1.5, after adjustment for age, BMI, waist circumference, and physical activity) among boys aged 11–14 years compared to the referent group sleeping 9 to 10 hours, but no such association was observed among girls of similar age [3]. Meanwhile, in the current study, after adjustment for age, BMI, physical activity, and smoking, statistically significant associations were found between short sleep durations (7– < 8 h/day and <7 h/day) and high BP (both prehypertension and hypertension) in both sexes separately. Another study in the United Stated investigated associations of the duration and quality of sleep with an elevated BP using wrist actigraphy in the sample of 238 adolescents aged 13–16 years [23]. The results of the above-mentioned study revealed that short sleep duration (≤6.5 hours) was significantly associated with a 2.8-fold increased odds of prehypertension in the unadjusted analysis, but this association was not significant in the adjusted analysis (after adjustment for sex, BMI percentile, and socioeconomic status) [23]. However, several studies have found no significant associations between short sleep duration (parent- or self-reported) and high BP in children and adolescents of various age groups: 3–10 years [24], 5–10 years [3], 10–12 years [26], 10–18 years [27], and 16–19 years [25]. Also, contrary to our findings, in a study of Portuguese adolescents aged 13 years, a significant association was found between high BP and long sleep duration in girls (>8.5 and <9.5 hours: aOR = 1.56; ≥9.5 hours: aOR = 1.83) compared with those sleeping ≤8.5 hours, but no significant association was found in boys [5].

The possible mechanisms underlying the association between insufficient sleep and the risk of high BP are uncertain. However, several studies have suggested that insufficient sleep increases the activity of the sympathetic nervous system (it is one of the mechanisms explaining the association) [37-39]. Other mechanisms may include an activation of the renin-angiotensin system and an enhanced production of the vasoconstrictor endothelin [40]. It has been also reported that sleep deprivation may cause changes in the immune, the metabolic, and the endocrine systems, which can be associated with an increased risk of cardiometabolic diseases [41].

Our study has several limitations. In this research, sleep duration was self-reported, and was not analyzed separately by weekdays and weekends. Further studies are required to investigate the associations of the duration and quality of sleep using objective measurements of sleep (such as actigraphy) with an increased BP among children and adolescents. However, in larger epidemiological studies, information on sleep duration in children and adolescents is typically obtained by subjective measures (self-report and parent-report measures), which are simple and cost-effective methods. Current smoking and physical activity were self-reported in the present study as well. Not all study subjects answered questions about the analyzed risk factors in sufficient detail, and thus these subjects were excluded from the analysis; however, there were no significant differences in the characteristics including age, sex, BMI, weight, height, and BP levels between the subjects who were excluded from the analysis and the included subjects. The current study examined only a sample of 12–15 year-old children and adolescents of Kaunas city and Kaunas district (Lithuania). Further research is also needed to examine samples of younger children and older adolescents in Lithuania. Furthermore, in the present study, all BP readings were obtained by an automatic oscillometric BP monitor, although, according to the Fourth Report, an elevated BP reading obtained with an oscillometric device should be repeated by using auscultation [32].

Despite the above-mentioned limitations, the results of the current study emphasized a high prevalence of elevated BP and a high prevalence of short sleep duration among Lithuanian schoolchildren, and indicated that short sleep duration was associated with high BP. Sufficient sleep duration in children and adolescents aged 12–15 years is an essential factor in the prevention of high BP. Therefore, public health strategies need to focus more on the prevention and control of modifiable risk factors associated with the development of cardiovascular diseases.

## Conclusions

Our results showed a high prevalence of prehypertension (12.6%) and hypertension (22.5%), and also a high prevalence of short sleep duration (<8 h/day: 29.7%) among 12–15 year-old children and adolescents living in Kaunas city and Kaunas district. Short sleep durations (7– < 8 h/day and <7 h/day) were significantly associated with increased odds of prehypertension and hypertension among Lithuanian schoolchildren. The data of our study may indicate possible interventions in families, including advice for parents to control children’s and adolescents’ sleep duration. Moreover, further prospective studies are needed to confirm these findings. If confirmed in future prospective studies, the findings of the present study may have implications for assessing sleep in children and adolescents to reduce the burden of cardiovascular disease.

## Abbreviations

aOR: adjusted odds ratio; BMI: Body mass index; BP: Blood pressure; CI: Confidence interval; DBP: Diastolic blood pressure; OR: Odds ratio; SBP: Systolic blood pressure; SD: Standard deviation.

## Competing interests

The authors declare that they have no competing interests.

## Authors’ contributions

RK contributed to writing the manuscript and the analysis and interpretation of the data. VD contributed to the study concept and design, writing the manuscript, and the analysis of the data. Both authors read and approved the final manuscript.

## Pre-publication history

The pre-publication history for this paper can be accessed here:

http://www.biomedcentral.com/1471-2458/14/255/prepub
